# Diagnosis in Stricture of the Œsophagus

**Published:** 1892-06-18

**Authors:** 


### SURGERY.

### DIAGNOSIS IN STRICTURE OF THE ŒSOPHAGUS.

When we investigate the numerous ways in which these strictures are produced our first feeling is one of astonishment at their comparative rarity. We should naturally expect to find very frequent cases where accidental injuries had caused the occlusion of a tube in which such great force is constantly exerted. We should imagine that the narrow passage would often be blocked by syphilitic growths, by innocent and malignant tumours, or compressed by changes in the organs surrounding it; and, finally, we might look for many instances where the motor mechanism had failed and could no longer fulfil its functions. Indeed, as regards the rest of the alimentary canal, all these affections abound, and many of us are throughout our lives taking remedies to overcome some minor forms of irregularity, such as constipation or diarrhœa. Yet the œsophagus does its work from day to day, and is hardly ever complained of unless a malignant or incurable disease causes a complete and final breakdown. Its diseases are rare and few in number, but the difficulty of distinguishing between them during life is often insurmountable. We may employ auscultation, the œsophagoscope, or bougies in vain, unless we give the closest attention to the state of all the neighbouring organs. Mackenzie insists on the importance of employing chloroform in making a careful examination with bougies, and on the great skill and practice needed for the interpretation of sounds heard through the stethoscope along the left side of the dorsal vertebræ.

The symptom of stricture which is most usually prominent is difficulty of swallowing. This may be due to many causes external to the œsophagus, as well as to its own diseases. Tumours in the pharynx, larynx, or the thorax, such as aneurisms, displacements of the spinal column, and foreign bodies which have become impacted in the tube itself, may cause it, as well as affections of the œsophagus, so that we often find extreme difficulty in localising the seat of the mischief. The forms of stricture, properly so called, are the spasmodic, the fibrous or cicatricial, and those produced by new growths. Malignant growths are nearly always carcinomata, very rarely sarcomata. Erichsen remarks that the commonest type is epithelioma, situated high up immediately behind the larynx, while at the lower end scirrhous may extend upwards from the stomach. Dr. Newman, in his recent lectures, holds that any part of the tube may be affected by epithelioma, and that the favourite spots are those on a level with the cricoid and with the bifurcation of the trachea. The growth may ulcerate away at an early stage, affording some relief to the dysphagia, but soon contraction follows, with rapid increase of difficulty in swallowing. The higher the situation, the more rapid is the occurrence of dysphagia, and the greater the danger of interference with the respiration. When, however, we have to do with one at the level of the bifurcation of the trachea, interference with swallowing comes on later, but a new risk, that of ulceration into one of the great vessels, becomes prominent. The difficulty of diagnosis from aneurism at this place is often considerable, and the danger to be faced in passing a bougie is porportionately increased. There are, indeed, cases where it is impossible to determine the real affection. There may be no enlarged glands, no vomiting of blood from ulcerated surfaces, and even no pain. The thoracic organs may appear to be perfectly normal, the apparent cachexia may be accounted for by the loss of blood and the mental distress. In some of these instances a valuable symptom of aneurism is afforded by the paralysis of the left vocal chord. Occasionally this may be produced by other diseases, but in the great majority of cases aneurism turns out to be the reason for its occurrence. Again, a tugging of the trachea when held tense by the fingers may be found in some aneurisms. The tumours which spring from the lower end of the œsophagus have more room for growth before they entirely stop the passage of food. The dysphagia may be at first slight and intermittent, but gradually increases. A scirrhous growth, again, may remain stationary for a long period, while epithelioma, and still more adenoid, spread rapidly. If, too, the œsophagus is attacked low down dilatation may take place above, and the food will regurgitate some time after it has been swallowed. The contents of the tube, as Dr. Newman remarks, simply well up, as in cerebral vomiting, with out retching, but tend to become offensive and blood-stained, as ulceration occurs. Innocent tumours are chiefly remarkable from their great rarity, and can only be diagnosed from malignant ones, aneurism and traumatic stricture, by their slow progress and the absence of more serious indications.

Strictures caused by cicatrices following injury, such as those produced by drinking acids, are usually marked by a history of some accident with violent pain and inability to swallow, which passes off in a few days. After some weeks of complete relief, contraction gradually comes on, and at length entirely obstructs the passage. Though benefit results from treatment by bougies, spasm often arises when they are introduced into these strictures. Here, again, in default of a clear history, we have to rely for diagnosis on the absence of enlarged glands, of pain, and of blood-stained vomit, and possibly on the age of the patient.

Pure spasmodic stricture is found chiefly in women of a gouty or nervous temperament, but may be met with in men. The patients are generally able to take a fair amount of food, and are not emaciated. The spasm comes on towards the end of a meal, especially if they are in company. A bougie can be introduced by firm pressure, and when this is relaxed the instrument is often driven out with force. As a starting-point for the spasm, there is often some superficial ulceration or chronic inflammation in the pharynx, near to which the constriction takes place, though the spot where a bougie is met by resistance may vary from day to day.

The symptoms produced by a syphilitic growth may resemble either those of an ulcerating cancer, or the pressure effects of a thoracic tumour. The history and the effect of a course of iodide is often the only means of diagnosis. However, the affection is rarely met with, the œsophagus almost always escaping even in the most widely-spread attacks.

Mackenzie points out that we may easily mistake cancer of the pylorus, when accompanied by vomiting, for malignant disease of the œsophagus, unless we note that there is an acid reaction in matter returned after an hour or more from the stomach, while that rejected from a dilated œsophagus is usually alkaline or neutral. There may be in either case an absence of hydrochloric acid and of digestive changes. The varicose veins of the lower end of the gullet which occur as a natural effort to restore the circulation in cirrhosis of the liver, rarely, if ever, cause obstruction. There may be some uneasy sensations or the disappearance of existing ascites as the veins enlarge, followed by serious hæmorrhage.

To recapitulate, in cancer we have progressive dysphagia, vomiting of a blood-stained fluid more or less frothy and mucopurulent, the obstruction coming on sooner the higher the situation of the growth. Bougies reveal a block in the tube at a fixed spot, and there is the absence of symptoms of other disease in the thoracic organs. Cachexia, pain, and enlarged glands are usually present. Innocent tumours grow very slowly. In traumatic lesions the accident, the recovery, and the subsequent obstruction must be noted, together with the age of the patient. Syphilitic cases are indistinguishable apart from their history and results, while in spasmodic ones the absence of emaciation the character of the patient, and the result of examination by bougies, are to be considered.

